# Computerized tomography‐derived body composition metrics are associated with 24‐h urine lithogenic parameters

**DOI:** 10.1002/bco2.70152

**Published:** 2026-01-15

**Authors:** Reza Lahiji, Lorenzo Storino Ramacciotti, Ernie Morton, Edouard H. Nicaise, Adam Braunschweig, Gregory Palmateer, Benjamin Schmeusser, Dattatraya Patil, Maxwell Richardson, Frank Glover, Ethan Kearns, Aaron Lay, Mohammad Hajiha, Viraj A. Master, Kenneth Ogan

**Affiliations:** ^1^ Department of Urology Emory University School of Medicine Atlanta GA United States; ^2^ Department of Urology Lenox Hill Hospital New York New York USA; ^3^ Department of Urology University of Indiana School of Medicine Indianapolis Indiana USA; ^4^ Winship Cancer Institute Emory University School of Medicine Atlanta Georgia USA

**Keywords:** body composition, kidney stones, skeletal muscle, subcutaneous adiposity, urine analysis, visceral adiposity

## Abstract

**Background:**

The relationship between body composition and lithogenic urine parameters remains poorly defined. This study aimed to evaluate associations between computerized tomography (CT)‐derived body composition metrics and 24‐h urine findings.

**Methods:**

Stone‐forming patients in our Nephrolithiasis Database who underwent 24‐h urine testing and CT within 120 days were retrospectively reviewed. Skeletal muscle index (SMI), visceral adipose tissue index (VATI), subcutaneous adipose tissue index (SATI) and skeletal muscle density (SMD) were calculated from segmented L3 axial images. Spearman correlations and multivariable logistic regression tested associations between body composition and 24‐h urine markers.

**Results:**

Among 443 patients, all body composition metrics demonstrated numerous correlations with 24‐h urine marker values on Spearman analysis. After adjusting for confounders, higher SMI quartiles were associated with increased odds of elevated urine volume (OR 2.13–2.71), hyperoxaluria (OR 2.11), hyperuricosuria (OR 2.60) and hypernatriuria (OR 2.70). Higher VATI was associated with reduced odds of elevated urine volume (OR 0.44), SATI with elevated sodium excretion (OR 2.35–2.38) and higher SMD with decreased odds of elevated oxalate (OR 0.50) and hypocitraturia (OR 0.41).

**Conclusions:**

CT‐derived body composition metrics show distinct and clinically meaningful relationships with 24‐h urine profiles. Muscle mass, adipose distribution and muscle quality each influence lithogenic risk, supporting incorporation of body composition assessment into metabolic evaluation of stone‐forming patients.

## 1 INTRODUCTION

For millennia, urine has served as a diagnostic mirror into the human body's internal physiology, reflecting both health and disease.^1^ As a metabolic by‐product, the composition of urine is influenced by the interplay of systemic processes, dietary intake and physiological states.^1^ Renal stones typically form from the nucleation of urine composite crystals once supersaturation levels have been reached and are varied in structure depending on their constituents. Complex interactions between promoters and inhibitors of crystallization in the urine play a role in the development of nephrolithiasis. Literature examining the relationship between said constituents and body composition is scarce, with the majority of the literature investigating broad renal stone associations rather than specific urine composition metrics.^2^ While body composition has been assessed through various methods (i.e., body mass index [BMI], waist–hip ratio, skinfold callipers, bioelectrical impedance [BIA] and dual X‐ray absorptiometry [DEXA] scans), current tools are widely inconsistent and impractical for daily clinical use.^3^, ^4^, ^5^ Non‐contrast computerized tomography (CT) remains the most practical method in establishing body composition metrics due to its ubiquitous use in renal stone imaging.^6^ When assessing urine composition, a 24‐h collection and urinalysis remains the gold standard in high‐risk patients with recurrent nephrolithiasis and metabolic pathologies as outlined by various guidelines.^7^ With the rising incidence of nephrolithiasis, identifying risk factors associated with stone formation is imperative.^8^ This study aims to characterize, if present, the relationship between CT‐derived body composition metrics and 24‐h urine profiles.

## 2 METHODS

### 2.1 Patient selection

This study was approved by the Institutional Review Board at our institution. Our prospectively maintained nephrolithiasis database was reviewed. Patients diagnosed with nephrolithiasis between 2010 and 2022 were eligible for inclusion if they had undergone both a 24‐h urine evaluation using LithoLink and CT imaging within 120 days of urine collection. An interval of 120 days was selected to improve data robustness while maintaining relative body composition similarity. Patients with incomplete data on urine capture and/or lacking CT imaging were excluded.

### 2.2 Patient characteristics and data collection

Patient demographics collected included age, race, BMI, comorbidities and pharmacologic agents. Comorbidities recorded included diabetes mellitus, hypertension, chronic kidney disease (CKD), gastroesophageal disease and hyperlipidaemia. Pharmacologic information collected included various medications, alkalinizing agents and thiazide diuretics.

**FIGURE 1:**
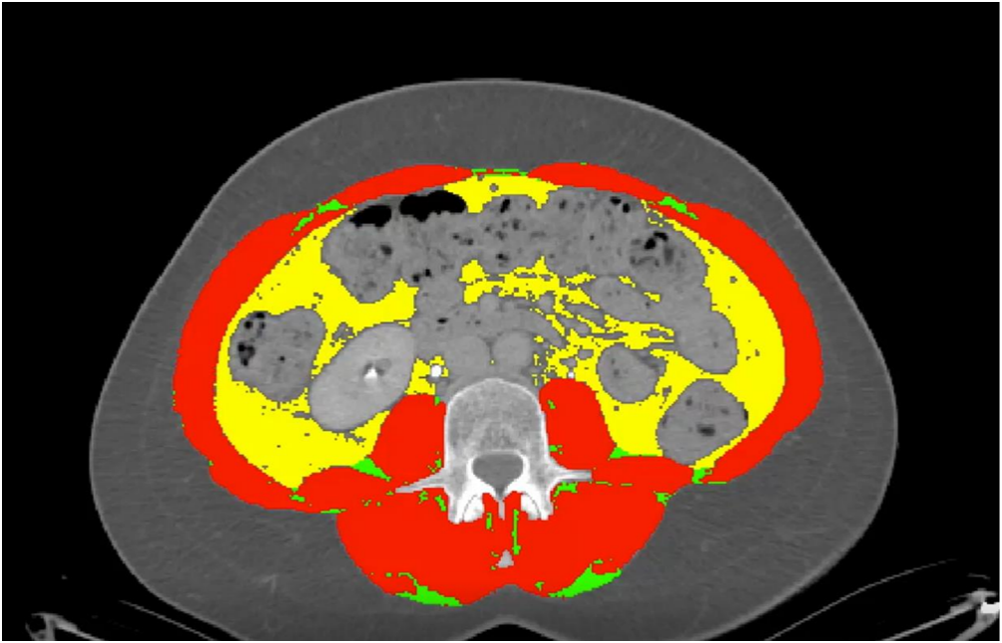
Axial CT slice at the mid‐L3 vertebral level segmented using Slice‐O‐Matic software and Horos, taken from Steele et al. guidance.^9^ *Note*: Visceral fat is marked as yellow, while muscle groups are marked as red and connective tissue as green.

Body composition was then assessed using axial CT images at the mid‐L3 vertebral level using Slice‐O‐Matic software (TomoVision, Quebec, Canada) and Horos as described by Steele et al., (Figure 1).^9^ Different Hounsfield unit (HU) thresholds were then applied to delineate tissue for measurement. Axial cross‐sectional areas of the psoas, paraspinal muscle groups, obliques and rectus muscle groups were used to compute skeletal muscle index (SMI, cm^2^/m^2^) using HU thresholds of −29 to 150. Visceral adipose tissue index (VATI, cm^2^/m^2^) was then measured using HU thresholds of −150 to −50, ensuring no luminal contents or intra‐organ fat were measured. Subcutaneous adipose tissue index (SATI) was determined by delineating subcutaneous fat using HU thresholds of −190 to −30. Skeletal muscle density (SMD) was calculated using aggregate HUs across previously mentioned muscle groups to assess muscle quality.

Using LithoLink© product guidance, patients were instructed to collect their urine over a 24‐h period and return their samples within 4 days. Data points from 24‐h collections included total urine volume (Volume, L), supersaturation of calcium oxalate (SSCaOx), urinary calcium (Ca24, mg/day), urinary oxalate (Ox24, mg/day), urinary citrate (Cit24, mg/day), supersaturation of calcium phosphate (SSCaP), pH, supersaturation of uric acid (SSUA), uric acid (UA24, g/day), urinary sodium (Na24, mg/day), creatinine excretion (Cr24, /kg) and days to urine collection. Elevated urine output was defined as >2 L over a 24‐h period. Thresholds for abnormalities were defined per Litholink© cut‐offs, and prior 24‐h urine collection results were retrieved from our database.

### 2.3 Statistical analysis

Following data collection, patient demographics, clinical characteristics and body composition metrics were tabulated and comparative statistics (Wilcoxon rank sum and independent *t* tests) evaluated differences between sexes. Total cohort and sex‐specific Spearman correlations between body composition metrics and each of the 24‐h urine markers were calculated. Multivariable logistic models adjusted for SMI, VATI, SATI, SMD, age and race and evaluated associations with lithogenic abnormalities on 24‐h urine testing. Each body composition metric was assessed in conjunction with the others and stratified into quartiles, with the first quartile serving as the reference group. A significance threshold of *p* < 0.05 and two‐sided *p* values were used for the purposes of this study. Analyses were conducted using SAS software, Version 9.4 (SAS Institute Inc., Cary, NC, USA).

## 3 RESULTS

### 3.1 Cohort characteristics

A total of 433 patients met inclusion criteria, comprising 227 males and 206 females. Cohort characteristics are outlined in Table 1. The median (interquartile range [IQR]) age of our population was 57 (45–66) years. The participant racial distribution was 73% White, 18.2% Black and 8.2% other races. The median (IQR) BMI was 27.5 kg/m^2^ (24.2–32.4 kg/m^2^). Males were significantly more likely to have hypertension (51.6% vs. 38.2%, *p* = 0.006) and CKD (17.8% vs. 8.5%, *p* = 0.005) compared with females. Males were also more likely to be taking alkalinizing agents (13.1% vs. 6.5%, *p* = 0.03) and to have a Charlson comorbidity score of ≥1 (46.1% vs. 27.6%, *p* < 0.001). In addition, males had significantly higher SMI and VATI values (*p* ≤ 0.001) and were more likely to have hypercalciuria, hyperoxaluria, hyperuricosuria, hypernatremia and elevated urinary volume (>2 L) on 24‐h urine analysis compared with their female counterparts (all *p* < 0.05). Median (IQR) time from CT scan to urine collection was 49 (31–73) days.

**TABLE 1:** Demographics, clinical characteristics and body composition metrics according to sex.

Covariate	Total (*n* = 433)	Male (*n* = 227)	Female (*n* = 206)	*p* value
Age at collection (years)^a^	57 (45–66)	57 (46–68)	56 (45–65)	0.09
Volume (L)^a^	**1.9 (1.4–2.5)**	**2.0 (1.5–2.7)**	**1.7 (1.2–2.3)**	**<0.001**
SS CaOx^a^	5.6 (3.7–8.1)	5.5 (3.4–8.0)	5.8 (3.9–8.4)	0.16
Ca24 (mg/day)	164.0 (102.6–241.2)	174.3 (114.6–241.2)	152.4 (97.2–242.5)	0.052
Ox24 (mg/day)^a^	**34.8 (26.8–45.5)**	**38.7 (29.8–48.5)**	**31.4 (24.9–40.9)**	**<0.001**
Cit24 (mg/day)^a^	498.6 (303.0–733.3)	491.5 (306.4–751.8)	543.0 (299.1–731.3)	0.98
SSCaP^a^	0.71 (0.29–1.41)	0.64 (0.28–1.36)	0.74 (0.31–1.45)	0.27
pH^a^	6.02 (5.67–6.45)	6.00 (5.60–6.39)	6.04 (5.71–6.46)	0.14
SSUA	0.65 (0.23–1.35)	0.68 (0.27–1.44)	0.59 (0.20–1.22)	0.11
UA24 (g/day)	**0.58 (0.45–0.76)**	**0.65 (0.52–0.84)**	**0.51 (0.41–0.63)**	**<0.001**
Na24 (mg/day)	**151.3 (112.4–209.9)**	**167.8 (133.8–228.8)**	**134.9 (94.9–185.9)**	**<0.001**
Cr24/kg	**18.97 (15.32–22.69)**	**21.33 (17.83–24.29)**	**16.24 (13.3–19.73)**	**<0.001**
Days to collection	49 (31–73)	48 (31–72)	52 (30–78)	0.75
BMI (kg/m^2^)^a^	27.5 (24.2–32.4)	28.0 (24.9–31.9)	27.1 (23.8–33.0)	0.57
SMI (cm^2^/m^2^)^a^	**50.1 (42.6–57.3)**	**55.9 (49.6–61.8)**	**44.3 (39.4–49.7)**	**<0.001**
VATI (cm^2^/m^2^)^a^	**53.1 (27.8–80.0)**	**65.3 (42.0–88.7)**	**38.1 (21.6–64.1)**	**<0.001**
SATI (cm^2^/m^2^)^a^	**76.5 (52.5–111.0)**	**63.8 (46.7–88.8)**	**97.7 (67.2–131.9)**	**<0.001**
SMD (HU)^a^	**36.3 (30.2–42.3)**	**38.2 (32.2–44.3)**	**34.3 (27.2–40.6)**	**<0.001**
Accurate collection^b^	376 (86.8)	180 (87.4)	196 (86.3)	0.75
Diabetes^b^	83 (19.9)	50 (22.8)	33 (16.6)	0.11
Hypertension^b^	**189 (45.2)**	**113 (51.6)**	**76 (38.2)**	**0.006**
Chronic kidney disease^b^	**56 (13.4)**	**39 (17.8)**	**17 (8.5)**	**0.005**
Gastroesophageal disease^b^	111 (26.5)	54 (24.7)	57 (28.5)	0.37
Hyperlipidaemia^b^	**137 (32.7)**	**83 (37.9)**	**54 (27.0)**	**0.02**
Antihypertensives medication	47 (12.1)	21 (10.2)	26 (14.1)	0.23
Anti‐diabetic medication	101 (25.9)	56 (27.2)	45 (24.5)	0.54
Anti‐hyperlipidaemic medication^b^	32 (8.2)	20 (9.7)	12 (6.5)	0.25
Alkalinizing agent^b^	**39 (10.0)**	**27 (13.1)**	**12 (6.5)**	**0.03**
Thiazide diuretics	79 (20.3)	38 (18.5)	41 (22.3)	0.35
Charlson comorbidity ≥1	**156 (37.3)**	**101 (46.1)**	**55 (27.6)**	**<0.001**
Race				
White	**316 (73.0)**	**176 (77.5)**	**140 (68.0)**	**0.001**
Black^b^	**79 (18.2)**	**27 (11.9)**	**52 (25.2)**	
Other^b^	**38 (8.8)**	**24 (10.6)**	**14 (6.8)**	
CaOx primary composition^b^	**154 (70.7)**	**94 (77.0)**	**60 (62.5)**	**0.005**
≥2‐L urine volume	**198 (45.7)**	**121 (53.3)**	**77 (37.4)**	**0.001**
High SS CaOx (>10)^b^	57 (13.2)	29 (12.8)	28 (13.6)	0.8
High Ca24 (M: >250; F > 200)^b^	**121 (27.9)**	**53 (23.4)**	**68 (33.0)**	**0.025**
High Ox24 (>40)	**159 (36.7)**	**107 (47.1)**	**52 (25.2)**	**<0.001**
Low Cit24 (M: <450; F: <550)^b^	204 (47.1)	99 (43.6)	105 (51.0)	0.13
High SSCaP (>2)	54 (12.5)	22 (9.7)	32 (15.5)	0.07
Low pH (<5.8)	150 (34.6)	83 (36.6)	67 (32.5)	0.38
High SSUA (>1)	161 (37.2)	92 (40.5)	69 (33.5)	0.13
High UA24 (M: >0.8; F: >0.75)^b^	**96 (22.2)**	**69 (30.4)**	**27 (13.1)**	**<0.001**
High Na24 (>150)^b^	**220 (50.8)**	**143 (63.0)**	**77 (37.4)**	**<0.001**

*Note*: Statistically significant results are bolded.

Abbreviations: BMI, body mass index; Ca24, calcium per day; CaOx, calcium oxalate; CaP, calcium phosphate; Cit24, citrate per day; Cr24, creatinine per day; Na24, sodium per day; Ox24, oxalate per day; SATI, subcutaneous adipose tissue index; SMD, skeletal muscle density; SMI, skeletal muscle index; SS, supersaturation; UA, uric acid; VATI, visceral adipose tissue index.

^a^ Median (IQR).

^b^ Number (%).

### 3.2 Sex‐specific Spearman correlation calculations

Total and sex‐specific Spearman correlations are summarized in Table 2. Total cohort SMI was positively correlated with urinary volume (0.256), Ca24 (0.224), Ox24 (0.307), Cit24 (0.185), SSUA (0.179), UA24 (0.504) and Na24 (0.425) and negatively correlated with urinary pH (−0.135). Total cohort VATI was positively correlated with Ox24 (0.228), SSUA (0.270), UA24 (0.321) and Na24 (0.319) and negatively correlated with urinary pH (−0.256) and SSCaP (−0.129). Cohort SATI values were positively correlated with SSUA (0.162), UA24 (0.105) and Na24 (0.185) and negatively correlated with pH (−0.140). Finally, cohort SMD was positively correlated with urine SSCaOx (0.121), Ca24 (0.188), Cit24 (0.119), SSCaP (0.244) and pH (0.143).

**TABLE 2:** Total cohort and sex‐specific Spearman correlations between body composition and 24‐h urine markers.

	Covariates	Volume	SSCaOx	Ca24	Ox24	Cit24	SSCaP	pH	SSUA	UA24	Na24
Total cohort	SMI (cm^2^/m^2^)	**0.256 (<0.001)**	0.009	**0.224 (<0.001)**	**0.307 (<0.001)**	**0.185 (<0.001)**	−0.024	**−0.135 (0.005)**	**0.179 (<0.001)**	**0.504 (<0.001)**	**0.425 (<0.001)**
	VATI (cm^2^/m^2^)	0.122 (0.011)	0.022	0.088	**0.228 (<0.001)**	0.076	**−0.129 (0.007)**	**−0.256 (<0.001)**	**0.270 (<0.001)**	**0.321 (<0.001)**	**0.319 (<0.001)**
	SATI (cm^2^/m^2^)	−0.014	0.043	0.007	0.062	0.080	−0.065	**−0.140 (0.004)**	**0.162 (<0.001)**	**0.105 (0.029)**	**0.185 (<0.001)**
	SMD (HU)	−0.043	**0.121 (0.012)**	**0.188 (<0.001)**	−0.04	**0.119 (0.013)**	**0.244 (<0.001)**	**0.143 (0.003)**	−0.053	**0.164 (<0.001)**	0.017
Male	SMI (cm^2^/m^2^)	**0.263 (<0.001)**	0.042	**0.263 (<0.001)**	**0.140 (0.035)**	**0.283 (<0.001)**	0.071	−0.028	0.058	**0.361 (<0.001)**	**0.315 (<0.001)**
	VATI (cm^2^/m^2^)	0.072	−0.029	0.008	0.089	0.082	−0.097	**−0.161 (0.015)**	**0.160 (0.016)**	**0.182 (0.006)**	**0.285 (0.010)**
	SATI (cm2/m2)	0.110	−0.019	0.065	0.083	0.118	−0.012	−0.049	0.084	**0.235 (<0.001)**	**0.303 (<0.001)**
	SMD (HU)	−0.056	**0.141 (0.034)**	**0.220 (<0.001)**	−0.085	**0.153 (0.021)**	**0.262 (<0.001)**	**0.159 (0.017)**	−0.066	0.099	−0.043
Female	SMI (cm^2^/m^2^)	0.035	0.078	0.107	**0.246 (<0.001)**	0.067	−0.058	**−0.169 (0.015)**	**0.246 (<0.001)**	**0.416 (<0.001)**	**0.363 (<0.001)**
	VATI (cm^2^/m^2^)	0.042	0.096	0.099	**0.211 (0.002)**	0.025	**−0.154 (0.027)**	**−0.322 (<0.001)**	**0.353 (<0.001)**	**0.279 (<0.001)**	**0.238 (<0.001)**
	SATI (cm^2^/m^2^)	0.004	0.084	0.021	**0.259 (<0.001)**	0.042	**−0.174 (0.012)**	**−0.311 (<0.001)**	**0.342 (<0.001)**	**0.264 (<0.001)**	**0.341 (<0.001)**
	SMD (HU)	−0.119	0.119	0.107	−0.124	0.083	**0.257 (<0.001)**	**0.177 (0.011)**	0.126	0.098	−0.053

*Note*: Statistically significant results are bolded (*p* values in parentheses when statistical significance is present).

Abbreviations: Ca24, calcium per day; CaP, calcium phosphate; Cit24, citrate per day; Na24, sodium per day; Ox24, oxalate per day; SATI, subcutaneous adipose tissue index; SMD, skeletal muscle density; SMI, skeletal muscle index; SS, supersaturation; UA, uric acid; VATI, visceral adipose tissue index.

Among male patients, SMI was positively correlated with urinary volume (0.263), Ca24 (0.263), Ox24 (0.140), Cit24 (0.283), UA24 (0.361) and Na24 (0.315). VATI was positively correlated with SSUA (0.160), UA24 (0.182) and Na24 (0.285) and negatively correlated with urinary pH (−0.161). SATI was positively correlated with UA24 (0.235) and Na24 (0.303). Finally, SMD was positively correlated with SSCaOx (0.141), Ca24 (0.220), Cit24 (0.153), SSCaP (0.262) and pH (0.159).

Among female patients, SMI was positively correlated with Ox24 (0.246), SSUA (0.246), UA24 (0.416) and Na24 (0.363) and was negatively correlated with urinary pH (−0.169). VATI was positively correlated with Ox24 (0.211), SSUA (0.353), UA24 (0.279) and Na24 (0.238) and negatively correlated with SSCaP (−0.154) and urinary pH (−0.322). SATI was positively correlated with Ox24 (0.259), SSUA (0.342), UA24 (0.264) and Na24 (0.341) and negatively correlated with SSCaP (−0.174) and pH (−0.311). Finally, SMD was positively correlated with SSCap (0.257) and pH (0.177).

### 3.3 Multivariable logistic regression results

Multivariable regression results analysing associations between body composition and stone‐forming abnormalities on the 24‐h urine collection are detailed in Table 3.

**TABLE 3:** Multivariable logistic regression examining associations between body composition and stone‐promoting abnormalities on 24‐h urine testing.

Variable	High volume^a^	High SSCaOx^a^	High Ca24^a^	High Ox24^a^	High SSCaP^a^	High SSUA^a^	High UA24^a^	Low Cit24^a^	High Na24^a^	High pH^a^
SMI (cm^2^/m^2^)										
4th	**2.71 (1.38–5.34)**	0.98 (0.39–2.49)	1.53 (0.72–3.25)	**2.11 (1.07–4.16)**	1.34 (0.48–3.75)	1.24 (0.63–2.44)	**2.60 (1.08–6.26)**	0.67 (0.35–1.30)	**2.70 (1.35–5.41)**	**0.43 (0.22–0.85)**
3rd	**2.13 (1.16–3.93)**	0.69 (0.28–1.69)	1.42 (0.71–2.82)	1.02 (0.54–1.92)	1.42 (0.57–3.58)	1.04 (0.56–1.95)	1.84 (0.79–4.33)	0.65 (0.35–1.18)	1.59 (0.86–2.96)	**0.50 (0.27–0.94)**
2nd	1.45 (0.81–2.62)	0.96 (0.43–2.16)	1.38 (0.70–2.71)	1.15 (0.63–2.10)	1.28 (0.52–3.15)	1.00 (0.54–1.83)	1.58 (0.68–3.65)	0.64 (0.36–1.14)	1.12 (0.61–2.04)	0.66 (0.37–1.19)
1st	Reference	Reference	Reference	Reference	Reference	Reference	Reference	Reference	Reference	Reference
VATI (cm^2^/m^2^)										
4th	**0.44 (0.20–0.98)**	1.28 (0.41–3.98)	1.20 (0.49–2.90)	0.79 (0.36–1.77)	0.61 (0.18–2.06)	1.49 (0.67–3.30)	2.47 (0.91–6.72)	0.85 (0.40–1.79)	1.69 (0.75–3.80)	0.53 (0.25–1.14)
3rd	0.72 (0.37–1.39)	1.44 (0.57–3.64)	1.21 (0.57–2.57)	0.88 (0.45–1.74)	0.88 (0.33–2.33)	1.44 (0.73–2.86)	1.10 (0.46–2.65)	0.82 (0.43–1.57)	1.00 (0.51–1.98)	0.54 (0.28–1.03)
2nd	0.87 (0.47–1.61)	1.38 (0.58–3.30)	1.14 (0.56–2.33)	0.93 (0.49–1.77)	0.65 (0.25–1.67)	1.09 (0.56–2.09)	1.29 (0.57–2.95)	0.60 (0.32–1.10)	1.22 (0.64–2.32)	0.64 (0.35–1.15)
1st	Reference	Reference	Reference	Reference	Reference	Reference	Reference	Reference	Reference	Reference
SATI (cm^2^/m^2^)										
4th	1.16 (0.54–2.46)	0.64 (0.21–1.97)	1.21 (0.53–2.76)	1.26 (0.58–2.72)	0.69 (0.22–2.13)	2.05 (0.94–4.47)	2.01 (0.76–5.28)	0.85 (0.40–1.79)	**2.38 (1.09–5.23)**	0.70 (0.34–1.43)
3rd	1.11 (0.57–2.14)	1.02 (0.40–2.58)	1.41 (0.69–2.87)	0.78 (0.39–1.54)	1.05 (0.42–2.67)	1.18 (0.59–2.37)	1.65 (0.68–3.97)	0.68 (0.35–1.31)	**2.35 (1.18–4.65)**	0.86 (0.46–1.59)
2nd	0.71 (0.39–1.31)	0.95 (0.41–2.23)	0.55 (0.27–1.14)	0.73 (0.39–1.37)	0.38 (0.14–1.03)	1.38 (0.73–2.59)	1.62 (0.71–3.71)	0.74 (0.41–1.35)	1.07 (0.57–2.00)	0.80 (0.44–1.47)
1st	Reference	Reference	Reference	Reference	Reference	Reference	Reference	Reference	Reference	Reference
SMD (HU)										
4th	**0.45 (0.21–0.95)**	1.28 (0.44–3.78)	1.35 (0.60–3.06)	0.62 (0.29–1.32)	0.78 (0.27–2.25)	1.05 (0.49–2.25)	1.34 (0.53–3.34)	**0.41 (0.19–0.86)**	0.77 (0.36–1.67)	1.15 (0.54–2.43)
3rd	0.71 (0.38–1.32)	1.45 (0.59–3.53)	1.49 (0.74–3.01)	**0.50 (0.26–0.95)**	0.59 (0.22–1.58)	1.29 (0.69–2.41)	1.25 (0.57–2.75)	0.58 (0.31–1.07)	0.60 (0.32–1.16)	1.02 (0.53–1.98)
2nd	0.92 (0.50–1.67)	1.03 (0.41–2.56)	1.45 (0.74–2.86)	0.71 (0.39–1.30)	0.60 (0.23–1.58)	1.05 (0.57–1.93)	1.38 (0.64–2.97)	0.60 (0.33–1.09)	1.03 (0.54–1.93)	1.25 (0.68–2.30)
1st	Reference	Reference	Reference	Reference	Reference	Reference	Reference	Reference	Reference	Reference
Age^b^	1.01 (0.99–1.02)	1.00 (0.98–1.02)	0.98 (0.97–1.00)	1.01 (0.99–1.02)	**0.97 (0.95–0.99)**	1.01 (1.00–1.03)	**0.96 (0.94–0.98)**	0.99 (0.97–1.00)	0.99 (0.97–1.00)	0.99 (0.97–1.00)
Race										
Black	**0.44 (0.25–0.77)**	1.99 (1.00–3.94)	**0.50 (0.26–0.96)**	0.90 (0.52–1.56)	0.66 (0.27–1.60)	0.82 (0.47–1.42)	0.77 (0.39–1.53)	1.63 (0.96–2.77)	0.62 (0.36–1.09)	1.27 (0.73–2.21)
Other	0.66 (0.27–2.68)	0.83 (0.27–2.53)	0.52 (0.22–1.21)	1.22 (0.59–2.54)	0.52 (0.16–1.63)	0.79 (0.36–1.71)	0.79 (0.31–1.99)	1.15 (0.57–2.34)	**2.30 (1.08–4.90)**	1.35 (0.65–2.80)
White	Reference	Reference	Reference	Reference	Reference	Reference	Reference	Reference	Reference	Reference

*Note*: Statistically significant values (*p* < 0.05) are bolded.

Abbreviations: Ca24, calcium per day; CaP, calcium phosphate; Cit24, citrate per day; HU, aggregate Hounsfield unit; Na24, sodium per day; Ox24, oxalate per day; SATI, subcutaneous adipose tissue index; SMD, skeletal muscle density; SMI, skeletal muscle index; SS, supersaturation; UA, uric acid; VATI, visceral adipose tissue index.

^a^ Odds ratios with 95% confidence intervals listed.

^b^ Age evaluated as continuous variable in this model.

Compared with patients in the first quartile of SMI values, patients in the third quartile had 2.13‐fold higher odds of elevated 24‐h urine volume (OR 2.13, 95% CI 1.16–3.93, *p* < 0.05). Similarly, patients with SMI values in the fourth quartile had 2.71‐fold and 2.11‐fold higher odds of elevated 24‐h urine volume (OR 2.71, 95% CI 1.38–5.34, *p* < 0.05) and hyperoxaluria (OR = 2.11, 95% CI 1.07–4.16, *p* < 0.05), respectively. Patients with SMI values in the fourth quartile had 2.6‐fold (OR 2.6, 95% CI 1.08–6.26, *p* < 0.05) and 2.7‐fold (OR 2.7, 95% CI 1.35–5.41, *p* < 0.05) higher odds of hyperuricosuria and hypernatriuria, respectively. Finally, patients with SMI values in the third and fourth quartiles had a 0.50‐fold (OR 0.50, 95% CI 0.27–0.94, *p* < 0.05) and 0.43‐fold (OR 0.43, 95% CI 0.22–0.85, *p* < 0.05) the risk of elevated urine pH on 24‐hr urine collections, respectively. When analysing VATI correlations, patients in the fourth quartile had 0.44‐fold the odds of having elevated urine volumes as compared with patients in the first quartile (OR 0.44, 95% CI 0.21–0.95, *p* < 0.05). No other independent associations were observed with VATI.

Patients with SATI values in the third and fourth quartiles had 2.35‐fold (OR 2.35, 95% CI 1.18–4.65, *p* < 0.05) and 2.38‐fold (OR 2.38, 95% CI 1.09–5.23, *p* < 0.05) higher odds of elevated Na24, respectively; no other independent associations with SATI were observed.

Patient SMD values in the fourth quartile were associated with 0.45‐fold (OR 0.45, 95% CI 0.21–0.95, *p* < 0.05) and 0.41‐fold (OR 0.41, 95% CI 0.19–0.86, *p* < 0.05) the odds of elevated urine volume and reduced Cit24, respectively. Furthermore, SMD values in the third quartile were independently associated with 0.50‐fold (OR 0.50, 95% CI 0.26–0.95, *p* < 0.05) the odds of high Ox24. No other independent associations with urine lithogenic markers were observed with SMD.

With age assessed as a continuous variable there were 3% and 4% lower odds of developing elevated SSCaP (OR 0.97, 95% CI 0.95–0.99, *p* < 0.05) and UA24 (OR 0.96, 95% CI 0.94–0.98, *p* < 0.05) values per 1‐year increase in age across the cohort.

Compared with White patients, Black patients were significantly less likely to develop elevated urine volumes (OR 0.44, 95% CI 0.21–0.95, *p* < 0.05) and hypercalciuria (OR 0.50, 95% CI 0.26–0.96, *p* < 0.05). Patients from ‘Other’ ethnic backgrounds were 2.3 times (OR 2.30, 95% CI 1.08–4.90, *p* < 0.05) more likely to develop elevated Na24 levels compared with White patients. All body composition metric quartile median (min–max) values and patient number distributions are detailed in Table S1.

## 4 DISCUSSION

In this study, we described the relationship between CT‐guided body composition metrics and 24‐h urine profiles among a racially diverse cohort of stone‐forming patients. SMI, VATI, SATI and SMI were found to be associated with a myriad of key urinary parameters on Spearman and multivariable analyses. In addition to our primary findings, meaningful sex‐specific trends were also noted as body composition metrics demonstrated distinct correlation patterns with urinary profiles in males as compared with females.

The link between SMI and elevated urine volume has not been reported to date. Given that an estimated 73% of muscle mass by weight is composed of water, it may be that individuals with more muscle mass possess greater total body water.^10^ However, whether this translates to increased urine output remains uncertain. Potential explanations include increased fluid intake among more physically active patients, increased total volume available for hyperfiltration (explaining the hypernatriuresis observed among patients with SMI in the fourth quartile) and increased osmotically active metabolic by‐product production.^11^, ^12^, ^13^

Patients with SMI values in the fourth demonstrated more than double the odds of developing elevated 24‐h urine oxalate levels compared with those in the first quartile. Our findings mirror those reported by Fargue et al. demonstrating correlations between urine oxalate levels and lean body mass.^14^ The endogenous synthesis of oxalate may explain this observation.^17^ As skeletal muscle contains hydroxyproline and vitamin C, they may be metabolized by the liver into oxalate, contributing to systemic and subsequently urine oxalate load.^14^ While patients demonstrated increased odds of raised 24‐h urine oxalate, no association was observed with oxalate supersaturation suggesting such increases may not necessarily translate to increased stone risk.

Patients in the highest quartile of SMI values were also more likely to develop hyperuricosuria on 24‐h collections. This finding, similar to oxalate, may be driven by the endogenous production of uric acid due to the purine‐rich composition of skeletal muscle and/or potentially by a protein‐rich diet among such individuals.^15^, ^16^

When analysing visceral fat (VATI) regression results, this study's findings align with prior research investigating obesity and metabolic syndrome as risk factors for lithogenesis.^17^ Patients in the fourth quartile of VATI were nearly half as likely to have raised urine volumes compared with those in the first quartile. Though we would expect raised lithogenic supersaturation profiles among patients producing less urine, this was not reflected in our results. Interestingly, patients with a SATI value in the third and fourth quartiles had more than double the odds of elevated urine sodium. While the association between adiposity and hypernatriuria is well documented in the literature, the correlation between superficial (rather than visceral) adiposity on imaging is novel.^18^ The observed hypernatriuria among such patients may be due to increased dietary sodium intake among those with unhealthy diets.^19^

While several potentially lithogenic associations were observed with SMI, our results suggest that muscle tissue quality (determined using SMD) may play a more significant protective role. Patients with SMD values in the third and fourth quartiles had half the odds of elevated 24‐h urine oxalate and hypocitraturia, respectively. Together, these findings may explain why elevated SMI values were not associated with supersaturation profiles as urine oxalate and urate are buffered by citrate. Irrespective, given the novel nature of such findings, further validation is warranted.

Age and race also emerged as risk modifiers, with increasing age correlating with reduced odds of supersaturation of calcium phosphate and uric acid in the urine and their respective stones.^20^ A multivariable regression model revealed a 3% and 4% decreased risk by year of elevated supersaturation of calcium phosphate and elevated 24‐h uric acid levels, respectively. It may be that this rise is driven by age‐related declines in urinary excretion and thus raised serum uric acid levels.^21^ This hypothesis warrants further investigation prior to its acceptance. Additionally, Black patients also had lower odds of hypercalciuria (OR) and raised urine volume (OR), mimicking results by Taylor and Curhan.^22^

Our study contributes to a growing body of research exploring systemic factors associated with nephrolithiasis. Previous studies have largely focused on BMI for body composition despite its inability to distinguish between muscle and adipose compartments or between visceral and subcutaneous adiposity.^23^ There are several limitations inherent to this retrospective single‐institution study such as residual confounders, inability to establish causality, limited generalizability and a majority White cohort. Similarly, given approximately 30% of patients were on preventative medications, this likely influenced our results yet improved generalizability. While we tried to control demographic and pharmacologic variables, our study lacked data on diet, hydration and physical activity, all of which could influence urine composition. Recognizing the limitations associated with 24‐h urine collections, our findings demonstrate that CT‐derived metrics may serve as practical surrogate markers and/or aid in risk stratification in the future. These metrics offer the advantage of being readily available and convenient for patients as they utilize imaging routinely obtained during clinical care; with the emergence of artificial intelligence technology, the time burden associated with body segmentation may be overcome.

## 5 CONCLUSION

In conclusion, our findings describe the relationship between various CT‐derived body composition metrics and urinary stone risk factors. By delineating specific muscle and fat compartments associated with lithogenic urine abnormalities, our findings support the integration of CT‐derived profiling into personalized nephrolithiasis prevention strategies. Of note, our study demonstrated novel findings suggesting that the quality of skeletal muscle determined using density (rather than just total mass/surface area) may play a role in the prevention of nephrolithiasis. Future studies should explore our findings further, using standardized dietary and hydration protocols.

## AUTHOR CONTRIBUTIONS

**Reza Lahiji:** Conceptualization; methodology; data curation; investigation; formal analysis; project administration; visualization; writing—original draft; writing—review and editing. **Dattatraya Patil:** Formal analysis; statistical analysis; methodology; validation; writing—review and editing. **Lorenzo Storino:** Investigation; data interpretation; validation; writing—review and editing. **Ernie Morton:** Investigation; data interpretation; validation; writing—review and editing. **Edouard H. Nicaise:** Investigation; data interpretation; visualization; writing—review and editing. **Adam Braunschweig:** Investigation; data interpretation; methodology; writing—review and editing. **Gregory Palmateer:** Data interpretation; validation; visualization; writing—review and editing. **Benjamin Schmeusser:** Data interpretation; validation; resources; writing—review and editing. **Maxwell Richardson:** Investigation; data interpretation; validation; writing—review and editing. **Frank Glover:** Investigation; data interpretation; validation; writing—review and editing. **Ethan Kearns:** Data interpretation; validation; writing—review and editing. **Aaron Lay:** Data interpretation; validation; writing—review and editing. **Mohammad Hajiha:** Data interpretation; validation; writing—review and editing. **Viraj A. Master:** Validation; supervision; resources; funding acquisition; writing—review and editing. **Kenneth Ogan:** Validation; supervision; resources; funding acquisition; writing—review and editing.

## CONFLICT OF INTEREST STATEMENT

The authors have no conflicts of interest to declare.

## GENERATIVE AI USE

The authors did not use generative AI or AI‐assisted technologies in the development of this manuscript.

## Supporting information


**Table S1:** SMI and VATI quartile values used in multivariable regression analysis.
