# Correction to ‘computerized tomography‐derived body composition metrics are associated with 24‐h urine lithogenic parameters’

**DOI:** 10.1002/bco2.70174

**Published:** 2026-02-16

Lahiji R , Ramacciotti LS , Morton E , Nicaise EH , Braunschweig A , Palmateer G , Schmeusser B , Patil D , Richardson M , Glover F , Kearns E , Lay A , Hajiha M , Master VA , Ogan K . Computerized tomography‐derived body composition metrics are associated with 24‐h urine lithogenic parameters. BJUI Compass. 2026;7(1): e70152. 10.1002/bco2.70152 41551326 PMC12805417

In Table 1, we noticed an error in the placement of a superscript ‘a’ or ‘b’ in the first column. The corrected table is presented below.

**TABLE 1:** Demographics, clinical characteristics and body composition metrics according to sex.

Covariate	Total (*n* = 433)	Male (*n* = 227)	Female (*n* = 206)	*p*‐value
Age at collection (years)^a^	57 (45–66)	57 (46–68)	56 (45–65)	0.09
Volume (L)^a^	**1.9 (1.4–2.5)**	**2.0 (1.5–2.7)**	**1.7 (1.2–2.3)**	**<0.001**
SS CaOx^a^	5.6 (3.7–8.1)	5.5 (3.4–8.0)	5.8 (3.9–8.4)	0.16
Ca24 (mg/day)^a^	164.0 (102.6–241.2)	174.3 (114.6–241.2)	152.4 (97.2–242.5)	0.052
Ox24 (mg/day)^a^	**34.8 (26.8–45.5)**	**38.7 (29.8–48.5)**	**31.4 (24.9–40.9)**	**<0.001**
Cit24 (mg/day)^a^	498.6 (303.0–733.3)	491.5 (306.4–751.8)	543.0 (299.1–731.3)	0.98
SS CaP^a^	0.71 (0.29–1.41)	0.64 (0.28–1.36)	0.74 (0.31–1.45)	0.27
pH^a^	6.02 (5.67–6.45)	6.00 (5.60–6.39)	6.04 (5.71–6.46)	0.14
SS UA^a^	0.65 (0.23–1.35)	0.68 (0.27–1.44)	0.59 (0.20–1.22)	0.11
UA24 (g/day)^a^	**0.58 (0.45–0.76)**	**0.65 (0.52–0.84)**	**0.51 (0.41–0.63)**	**<0.001**
Na24 (mg/day)^a^	**151.3 (112.4–209.9)**	**167.8 (133.8–228.8)**	**134.9 (94.9–185.9)**	**<0.001**
Cr24/kg^a^	**18.97 (15.32–22.69)**	**21.33 (17.83–24.29)**	**16.24 (13.3–19.73)**	**<0.001**
Days to collection^a^	49 (31–73)	48 (31–72)	52 (30–78)	0.75
BMI (kg/m2)^a^	27.5 (24.2–32.4)	28.0 (24.9–31.9)	27.1 (23.8–33.0)	0.57
SMI (cm2/m2)^a^	**50.1 (42.6–57.3)**	**55.9 (49.6–61.8)**	**44.3 (39.4–49.7)**	**<0.001**
VATI (cm2/m2)^a^	**53.1 (27.8–80.0)**	**65.3 (42.0–88.7)**	**38.1 (21.6–64.1)**	**<0.001**
SATI (cm2/m2)^a^	**76.5 (52.5–111.0)**	**63.8 (46.7–88.8)**	**97.7 (67.2–131.9)**	**<0.001**
SMD (HU)^a^	**36.3 (30.2–42.3)**	**38.2 (32.2–44.3)**	**34.3 (27.2–40.6)**	**<0.001**
‐‐‐‐‐‐‐‐‐‐‐‐‐‐‐‐‐‐‐‐‐‐‐‐‐‐‐‐‐‐‐‐‐‐‐‐‐‐‐‐‐‐‐‐‐‐‐‐‐‐‐‐‐‐‐‐‐‐‐‐‐‐‐‐‐‐‐‐‐‐‐‐‐‐‐‐‐‐‐‐‐‐‐‐‐‐‐‐‐‐‐‐‐‐‐‐‐‐‐‐‐‐‐‐‐‐‐‐‐‐‐‐‐‐‐‐‐‐‐‐‐‐‐‐‐‐‐‐‐‐‐‐‐‐‐‐‐‐‐‐‐‐‐‐‐‐‐‐‐‐‐‐‐‐‐‐‐‐‐				
Accurate collection^b^	376 (86.8)	180 (87.4)	196 (86.3)	0.75
Diabetes^b^	83 (19.9)	50 (22.8)	33 (16.6)	0.11
Hypertension^b^	**189 (45.2)**	**113 (51.6)**	**76 (38.2)**	**0.006**
Chronic kidney disease^b^	**56 (13.4)**	**39 (17.8)**	**17 (8.5)**	**0.005**
Gastroesophageal disease^b^	111 (26.5)	54 (24.7)	57 (28.5)	0.37
Hyperlipidemia^b^	**137 (32.7)**	**83 (37.9)**	**54 (27.0)**	**0.02**
Antihypertensives medication^b^	47 (12.1)	21 (10.2)	26 (14.1)	0.23
Antidiabetic medication^b^	101 (25.9)	56 (27.2)	45 (24.5)	0.54
Anti‐hyperlipidaemic medication^b^	32 (8.2)	20 (9.7)	12 (6.5)	0.25
Alkalinizing agent^b^	**39 (10.0)**	**27 (13.1)**	**12 (6.5)**	**0.03**
Thiazide diuretics^b^	79 (20.3)	38 (18.5)	41 (22.3)	0.35
Charlson comorbidity ≥1^b^	**156 (37.3)**	**101 (46.1)**	**55 (27.6)**	**<0.001**
Race				
White^b^	**316 (73.0)**	**176 (77.5)**	**140 (68.0)**	**0.001**
Black^b^	**79 (18.2)**	**27 (11.9)**	**52 (25.2)**	
Other^b^	**38 (8.8)**	**24 (10.6)**	**14 (6.8)**	
CaOx primary composition^b^	**154 (70.7)**	**94 (77.0)**	**60 (62.5)**	**0.005**
‐‐‐‐‐‐‐‐‐‐‐‐‐‐‐‐‐‐‐‐‐‐‐‐‐‐‐‐‐‐‐‐‐‐‐‐‐‐‐‐‐‐‐‐‐‐‐‐‐‐‐‐‐‐‐‐‐‐‐‐‐‐‐‐‐‐‐‐‐‐‐‐‐‐‐‐‐‐‐‐‐‐‐‐‐‐‐‐‐‐‐‐‐‐‐‐‐‐‐‐‐‐‐‐‐‐‐‐‐‐‐‐‐‐‐‐‐‐‐‐‐‐‐‐‐‐‐‐‐‐‐‐‐‐‐‐‐‐‐‐‐‐‐‐‐‐‐‐‐‐‐‐‐‐‐‐‐‐‐				
≥2 L Urine volume^b^	**198 (45.7)**	**121 (53.3)**	**77 (37.4)**	**0.001**
High SS CaOx (>10)^b^	57 (13.2)	29 (12.8)	28 (13.6)	0.8
High Ca24 (M: >250; F > 200; mg/day)^b^	**121 (27.9)**	**53 (23.4)**	**68 (33.0)**	**0.025**
High Ox24 (>40; mg/day)^b^	**159 (36.7)**	**107 (47.1)**	**52 (25.2)**	**<0.001**
Low Cit24 (M: <450; F: <550; mg/day)^b^	204 (47.1)	99 (43.6)	105 (51.0)	0.13
High SS CaP (>2)^b^	54 (12.5)	22 (9.7)	32 (15.5)	0.07
Low pH (<5.8)^b^	150 (34.6)	83 (36.6)	67 (32.5)	0.38
High SS UA (>1)^b^	161 (37.2)	92 (40.5)	69 (33.5)	0.13
High UA24 (M: >0.8; F: >0.75; g/day)^b^	**96 (22.2)**	**69 (30.4)**	**27 (13.1)**	**<0.001**
High Na24 (>150; mg/day)^b^	**220 (50.8)**	**143 (63.0)**	**77 (37.4)**	**<0.001**

*Note*: Statistically significant results are emboldened.

Abbreviations: BMI, body mass index; Ca24, calcium per day; CaOx, calcium oxalate; CaP, calcium phosphate; Cit24, citrate per day; Cr24, creatinine per day; Na24, sodium per day; Ox24, oxalate per day; SATI, subcutaneous adipose tissue index; SMD, skeletal muscle density; SMI, skeletal muscle index; SS, supersaturation; UA, uric acid; VATI, visceral adipose tissue index.

^a^ Median (IQR).

^b^ Number (%).

We apologize for this error.

