# Supplementary material for: Computerized tomography‐derived body composition metrics are associated with 24‐h urine lithogenic parameters
Source: BJUI Compass. 2026 Jan 15;7(1):e70152. doi: 10.1002/bco2.70152 (PMC12805417; doi:10.1002/bco2.70152)
Supplement: Supplementary file 1 — Table S1: SMI and VATI quartile values used in multivariable regression analysis. [file BCO2-7-e70152-s001.docx]

| **Supplemental Table 1:** SMI and VATI quartile values used in multivariable regression analysis. | | | |
| --- | --- | --- | --- |
| **Variable** |  | **Median value (Min-Max)** | **Total (N= 443)** |
| SMI (cm2/m2) | | |  |
|  | 1st Quartile | 38.2 (12.9-42.6) | 108 |
|  | 2nd Quartile | 46.4 (42.6-49.8) | 108 |
|  | 3rd Quartile | 53.8 (50.1-57.4) | 109 |
|  | 4th Quartile | 62.8 (57.6-115.6) | 108 |
| VATI (cm2/m2) | | |  |
|  | 1st Quartile | 16.2 (0.3-27.6) | 108 |
|  | 2nd Quartile | 40.2 (27.8-52.4) | 108 |
|  | 3rd Quartile | 65.3 (53.1-80.0) | 109 |
|  | 4th Quartile | 100.9 (80.3-266.3) | 108 |
| SATI (cm2/m2) |  |  |  |
|  | 1st Quartile | 40.2 (5.1-52.4) | 108 |
|  | 2nd Quartile | 64.6 (52.5-76.3) | 108 |
|  | 3rd Quartile | 91.7 (76.5-111.0) | 109 |
|  | 4th Quartile | 143.9 (111.4-275.5) | 109 |
| SMD (HU) |  |  |  |
|  | 1st Quartile | 24.9 (2.4-30.2) | 108 |
|  | 2nd Quartile | 33.4 (30.2-36.3) | 108 |
|  | 3rd Quartile | 39.4 (36.3-42.3) | 109 |
|  | 4th Quartile | 46.4 (42.4-265.8) | 108 |
| Abbreviations: SMI (skeletal muscle index), VATI (visceral adipose tissue index), N (number); SATI (subcutaneous adipose tissue index); SMD (skeletal muscle density); HU (aggregate Hounsfield units). | | | |
